# A Case Series of Powassan Meningoencephalitis in a Single Institution in Connecticut With One Case Resembling Herpes Simplex Virus (HSV) Meningoencephalitis on MRI

**DOI:** 10.7759/cureus.83373

**Published:** 2025-05-02

**Authors:** Anup Uprety, Tapasya Bhusal, Juan Crespo-Quezada, Felipe Carmona Pires, Christopher Morgan

**Affiliations:** 1 Internal Medicine, Danbury Hospital, Danbury, USA; 2 Internal Medicine, Connecticut Institute for Communities, Greater Danbury Community Health Center, Danbury, USA

**Keywords:** cognitive impairment, hsv encephalitis, meningoencephalitis, powassan virus, tick-borne illness

## Abstract

Powassan virus (POWV) is a single-stranded RNA virus that belongs to the genus *Orthoflavivirus*, which is transmitted to humans through the bite of infected deer tick (*Ixodes scapularis*), with peak transmission during summer and fall. Most patients are asymptomatic; those who develop symptoms usually present with flu-like symptoms or neuroinvasive disease, including encephalitis, meningoencephalitis, or aseptic meningitis. The gold standard test for diagnosis is serological testing. The treatment is usually supportive care. In this case series, we have reported two cases that presented with fever, generalized weakness, and changes in mentation, later diagnosed with POWV meningoencephalitis. The magnetic resonance imaging (MRI) findings in the first case were suggestive of herpes simplex virus (HSV), imposing a diagnostic challenge. Patients might end up with cognitive impairment, speech difficulties, headaches, imbalance, and ophthalmoplegia as long-term consequences, and both of our cases had cognitive impairment, and one case had weakness, requiring further neuropsychiatry evaluation, as well as physical therapy. Although cases of POWV encephalitis are very rare, these cases describe the importance of serological testing for POWV, especially in endemic areas, in patients with a known or unknown history of tick bite.

## Introduction

Powassan virus (POWV) is a single-stranded RNA virus that belongs to the genus *Orthoflavivirus* [1]. The primary hosts of POWV are small- and medium-sized mammals and rodents, with the primary vector being the *Ixodes* tick species. POWV is transmitted to humans through the bite of infected ticks, specifically the black-legged tick or deer tick (*Ixodes scapularis*), with peak transmission during summer and fall [2].

The incubation period of POWV is 7-34 days. Most of the patients are asymptomatic; those who do may develop flu-like illnesses, nausea, vomiting, or morbilliform rash or neuroinvasive diseases, including encephalitis, meningoencephalitis, or aseptic meningitis [3]. The case fatality rate is estimated to be between 10% and 15% with long-term sequelae such as cognitive and speech difficulties, imbalance and difficulty walking, spastic quadriplegia, ophthalmoplegia, and headaches in 50% of the survivors [2]. There are no specific routine laboratory investigations for the diagnosis. Cerebrospinal fluid (CSF) analysis shows pleocytosis, which can be either lymphocytic or polymorphonuclear predominant [4]. However, the gold standard for diagnosis remains serological testing, specifically immunoglobulin M (IgM) antibody testing utilizing the enzyme-linked immunosorbent assay (ELISA) and immunofluorescence antibody (IFA) [2]. Magnetic resonance imaging (MRI) findings show T2-weighted fluid-attenuated inversion recovery (T2/FLAIR) hyperintensities in the basal ganglia, brainstem, and thalamus, which are non-variable; however, imaging findings continue to be nonspecific [5]. The treatment of POWV meningoencephalitis remains supportive as there are currently no clinical studies supporting improved clinical outcomes with antiviral agents or vaccines [6].

Here, we present two cases of POWV meningoencephalitis. The first is a 79-year-old woman in whom the diagnosis was complicated by the MRI findings suggestive of herpes simplex virus (HSV) encephalitis. This case has been reported as per the Case Reports (CARE) 2020 criteria [7]. The second is a 77-year-old man who had a headache, fever, and non-focal weakness and was later diagnosed with serum analysis.

## Case presentation

A 79-year-old woman was referred from the primary care physician's (PCP) office for mild hyponatremia and concerns about worsening changes in mentation, generalized weakness, malaise, myalgia, fevers, and headaches for two weeks. One month prior to the presentation, she noted having multiple embedded ticks. She had traveled to the Cape Cod area in Eastern Massachusetts, which is one of the endemic areas for tick-borne illness. She then developed a patchy, itchy, and erythematous rash on both her torso and upper and lower extremities for which she was seen by a dermatologist and treated with cephalexin, which was later switched to doxycycline due to a lack of improvement.

On presentation to the emergency department, her vitals were within normal limits. She appeared lethargic. The skin examination was remarkable for scattered pustules on her torso, right flank, and right upper extremity. The neurological examination was otherwise normal.

Complete blood count (CBC), complete metabolic panel, and urinalysis were grossly unremarkable except for hyponatremia at 128 and mild leukocytosis with neutrophilic predominance at 10.2×10^9^/L. EKG showed normal sinus rhythm. Chest X-ray and CT of the head without contrast were negative for any acute findings.

She was empirically started on ceftriaxone, vancomycin, and acyclovir for suspected meningoencephalitis. Her sodium corrected appropriately with fluid restriction. Doxycycline was eventually added due to concerns for tularemia. A lumbar puncture was performed. CSF analysis was significant for an opening pressure of 22 cmH_2_O, a lymphocytic pleocytosis with elevated white blood cell count of 58/μL (74% lymphocytes), red blood cell counts of 6/μL, glucose count of 59 mg/dL, and an elevated protein count of 67 mg/dL, suggesting viral meningoencephalitis. Therefore, vancomycin and ceftriaxone were discontinued, and the patient was continued on acyclovir and doxycycline.

Eventually, CSF gram stain and culture, fungal culture, and CSF acid-fast bacilli (AFB) culture showed no growth. The CSF studies were negative for enterovirus, cryptococcal antigen, varicella, HSV 1/2, West Nile virus, Eastern and Western equine, La Crosse, and St. Louis encephalitis. Serology also came back negative for *Mycoplasma pneumoniae*, tularemia, Rocky Mountain spotted fever, syphilis, and N-methyl-D-aspartate (NMDA) antibodies. Urine culture and blood culture showed no growth. Other studies were negative to include SARS-CoV-2 (COVID-19), human immunodeficiency virus (HIV), *Leptospira* serology, and an arthropod panel investigating for *Anaplasma*, *Babesia*, and Lyme. A rheumatologic workup showed a mild positive antinuclear antibody (ANA) of 1:80 titer with speckled pattern with negative double-stranded DNA antibody, centromere antibody, chromatin antibody, Jo-1 antibody, ribosomal P antibody, ribonucleoprotein (RNP) antibody, Scl-70 antibody, anti-Smith antibody, and Sjögren syndrome antigen A (SS-A) and Sjögren syndrome antigen B (SS-B) antibodies.

**Figure 1:**
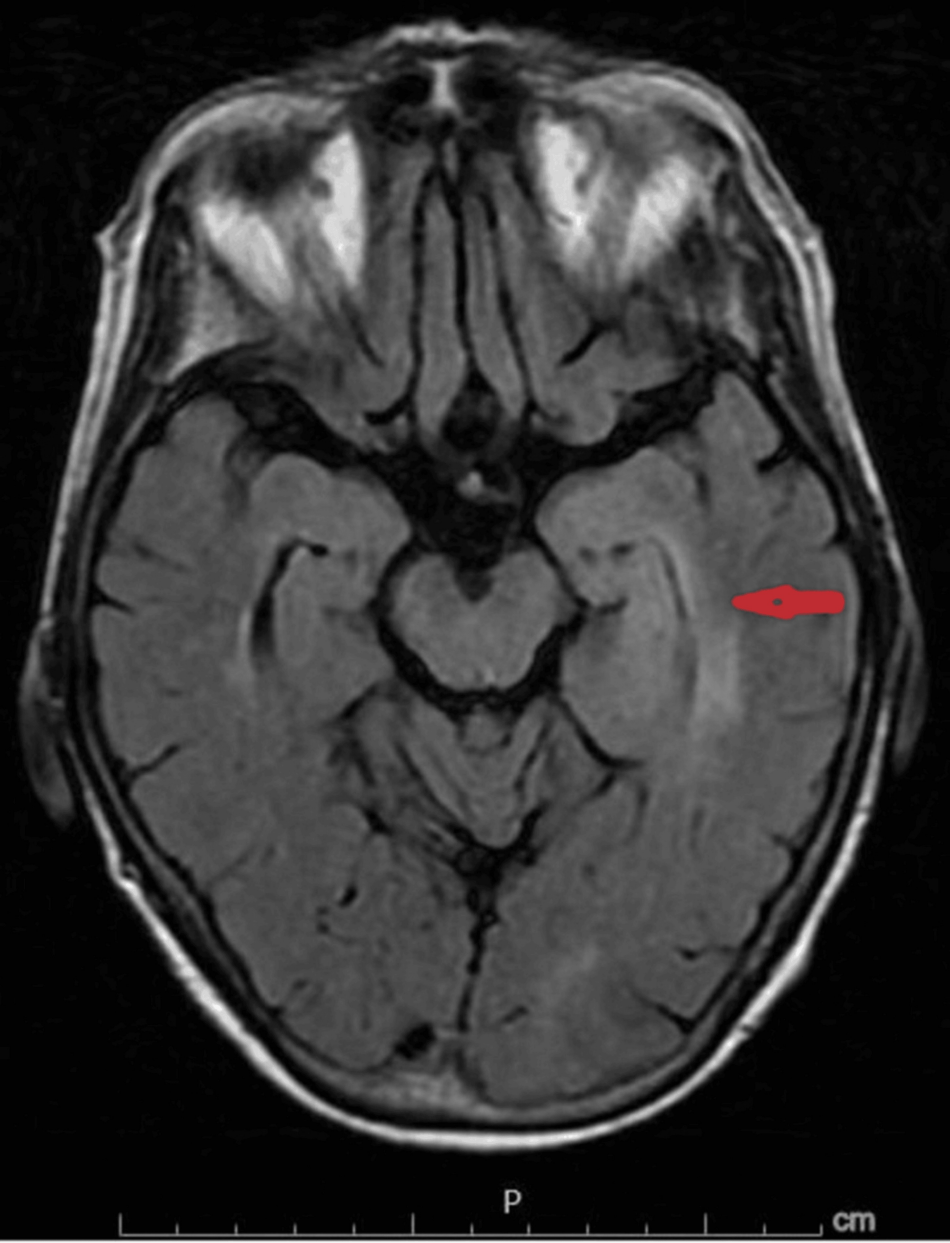
MRI of the brain with and without IV contrast shows mild asymmetric fullness and FLAIR hyperintensity about the medial left temporal lobe/hippocampus without postcontrast enhancement or midline shift. Findings are nonspecific and could relate to infectious etiologies such as herpes simplex encephalitis versus unilateral autoimmune encephalitis. MRI, magnetic resonance imaging; FLAIR, fluid-attenuated inversion recovery

The patient continued to have fluctuating mentation and fever during the hospital course. An MRI of the brain with and without contrast was obtained, which revealed mild asymmetric fullness and FLAIR hyperintensity about the medial left temporal lobe/hippocampus without postcontrast enhancement or midline shifts with concerns for infectious etiology such as herpes simplex encephalitis, an autoimmune process, or central nervous system (CNS) neoplasm or postictal changes (Figure 1). An electroencephalogram (EEG) revealed generalized background slowing consistent with cerebral dysfunction.

Considering her clinical presentation and the subsequent MRI findings on the temporal lobe, a decision was made to continue acyclovir and repeat lumbar puncture for HSV polymerase chain reaction (PCR). Due to concern for falsely negative HSV PCR in the early stages of the disease, lumbar puncture was repeated on the sixth day of the hospital stay, and the results were negative for HSV-1 and HSV-2 yet again. On the 10th day of hospitalization, Powassan virus IgM was positive, although PCR testing was negative. She continued to have cognitive impairment, but her mentation gradually improved. She is currently following up with her primary care provider and undergoing additional evaluation as an outpatient for cognitive impairment.

A 77-year-old man from Connecticut presented to the emergency department for an abrupt onset of fever, headaches, and vomiting for two days. He reported no tick bites.

Vital signs were within normal limits except for a fever of 38.8°C. He was alert and oriented to self, place, time, and purpose. The neurological examination was normal with negative meningeal signs. The skin examination revealed no rash.

His initial CBC and comprehensive metabolic panel (CMP) were unremarkable. PCR testing for Lyme, *Anaplasma*, and *Babesia* was negative. A COVID-19 PCR and respiratory viral panel were negative. Chest X-ray showed no evidence of acute cardiopulmonary disease.

The patient was started empirically on doxycycline and ceftriaxone. He remained febrile, and headaches persisted despite supportive care and treatment with antibiotics.

A CT scan of the chest, abdomen, and pelvis did not reveal any identifiable infectious focus. A lumbar puncture was then performed. The CSF analysis was clear and contained 21 nucleated cells with 90% lymphocytes. CSF protein was elevated at 88 mg/dL, and CSF glucose was normal. CSF gram stain and culture were negative, as well as blood and urine cultures. Acyclovir was started empirically for herpes simplex virus (HSV) coverage, and antibiotics were discontinued. Subsequent CSF PCR was negative for HSV and enteroviruses.

The fever resolved spontaneously, and he was discharged on a short course of steroids for headaches. CSF IgM and plaque reduction neutralization test against POWV sent out to the CDC returned positive 28 days after hospital discharge.

**Figure 2:**
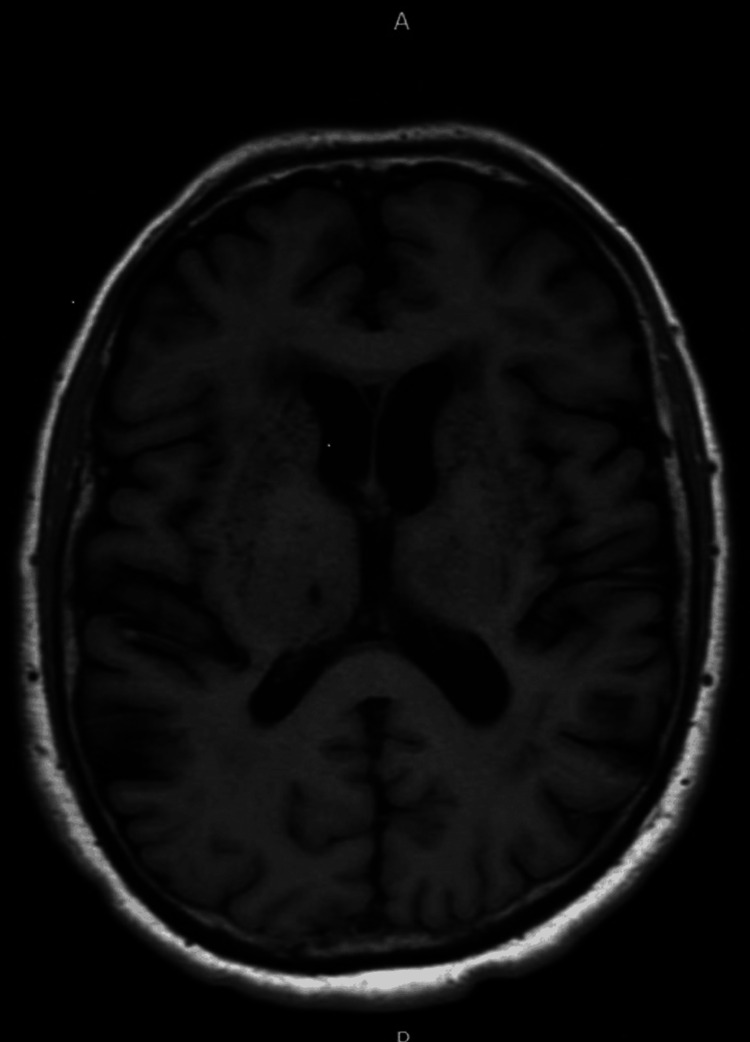
MRI of the brain without contrast with T1 attenuated sequence shows severe microvascular ischemic disease with several chronic lacunar infarcts bilaterally in the basal ganglia and in the right thalamus. MRI: magnetic resonance imaging

The patient continued to experience fatigue, weakness, memory impairment, intermittent headaches, and difficulty with ambulation that began after his POWV encephalitis diagnosis. Outpatient MRI showed severe microvascular ischemic disease with several lacunar infarcts in the basal ganglia, which were chronic findings without other findings suggesting encephalitis (Figure 2). Patient declined neuropsychiatric testing. He was referred to physical therapy with some improvement in weakness and balance.

## Discussion

POWV was first isolated in 1958 from the brain of a young boy who died of encephalitis in Powassan, Ontario [2]. Since then, POWV human cases were reported in the United States, Canada, and Russia. Around 40-45 human POWV cases were documented in North America from the 1950s to the mid-2000s. Around 290 POWV cases have been reported from 2004 to 2022, with most reported cases in Wisconsin, New York, Massachusetts, and Minnesota and 19 reported cases in Connecticut [8].

The virus is transmitted by tick bites, and risk factors for infection include outdoor activities in endemic areas, contact with wild animal hosts of POWV, and pets infested by ticks [2]. Most patients either are asymptomatic or usually present with the prodromal phase with nonspecific flu-like symptoms, such as fever, chills, malaise, generalized weakness, sore throat, headache, myalgia, nausea, and vomiting, which last for 1-3 days [9]. However, the proportion of patients who develop CNS infection remains unclear [10]. Neurological symptoms usually develop weeks to months after the initial prodromal illness and include headache, fever of >38°C, altered sensorium, hemiplegia, even quadriplegia, paresis, tremors, and focal palsies; less frequently, nystagmus, ophthalmoplegia, facial palsy, myelitis, hallucinations, and respiratory failure are also reported [9].

Both patients presented with fever, headaches, and generalized weakness in an endemic area for tick-borne illness. Interestingly, our first case also presented altered mentation and exposure to multiple embedded ticks. Both patients were first covered empirically with antibiotics. Initial CSF studies were not consistent with bacterial meningitis, and antimicrobials were narrowed to cover for viral etiologies. In addition, serological tests for other common arthropod-borne diseases were negative, further supporting the diagnosis of viral encephalitis.

The MRI findings in our first case were more suggestive of HSV encephalitis, which led to the diagnostic dilemma between Powassan virus encephalitis and HSV encephalitis, as both can present with similar clinical manifestations and CSF findings [11]. In addition, the patient had exposure to tick bites from the POWV-endemic areas. Negative HSV serology on the second lumbar tap and positive POWV IgM confirmed the diagnosis of POWV encephalitis similar to our second case.

The case definition for POWV-associated neuroinvasive infection includes clinical criteria (fever of 38°C with any peripheral or central nervous system dysfunction) and at least one of the following laboratory findings: (i) the direct detection of POWV in tissue, blood, or CSF by virus culture or a molecular detection method, (ii) a fourfold rise of POWV-specific antibody titers in paired serum samples, (iii) the presence of POWV-specific IgM in serum and the presence of POWV-neutralizing antibodies in the same specimen or a specimen collected during convalescence, and (iv) the presence of POWV-specific IgM in CSF and the simultaneous absence of IgM for other arboviruses endemic to the region where exposure occurred [2]. Our cases were diagnosed by exclusion and fulfilled the above diagnostic criteria. Though our patients showed improvement in mentation, they sustained cognitive impairment as a neurological sequela, underscoring the importance of adapting preventive measures such as tick education and prevention for those who live in or visit highly endemic areas [9].

## Conclusions

The diagnosis of POWV encephalitis can be challenging since the clinical presentation coincides with multiple other causes of encephalitis. Also, there is a diagnostic delay given the on-site availability of the test and the delay in getting test results. Our first case presents a diagnostic dilemma as the MRI findings partially resemble an HSV infection. Nevertheless, it is critical to address the underlying metabolic disorders and empirically treat any potential infections using a multidisciplinary approach until a definitive diagnosis is established. In addition, these cases highlight the importance of obtaining POWV serology in patients with unexplained fever, nonspecific symptoms, and headaches in an endemic area, especially for patients with a documented history of tick bites, and the need to make POWV testing more readily available. We need to raise more awareness regarding the progressive debilitating symptoms of the disease among clinicians so that proper follow-up visits and referrals are done.
